# Mn_3_SnN‐Based Antiferromagnetic Tunnel Junction with Giant Tunneling Magnetoresistance and Multi‐States: Design and Theoretical Validation

**DOI:** 10.1002/advs.202502985

**Published:** 2025-06-10

**Authors:** Shiqi Liu, Tingwei Chen, Baochun Wu, Haodong Fan, Yingmei Zhu, Sheng Bi, Yuntian Liu, Yinuo Shi, Wenbiao Zhang, Mengxi Wang, Qiang Li, Jie Yang, Jing Lu, Tiejun Zhou, Bo Liu

**Affiliations:** ^1^ State Key Laboratory of Spintronic Devices and Technologies Hangzhou 311305 P. R. China; ^2^ State Key Laboratory of Low Dimensional Quantum Physics and Department of Physics Tsinghua University Beijing 100084 P. R. China; ^3^ Research Center for New Materials Computing Zhejiang Lab Hangzhou 311100 P. R. China; ^4^ Department of Physics and Shenzhen Institute for Quantum Science and Engineering (SIQSE) Southern University of Science and Technology Shenzhen 518055 P. R. China; ^5^ Department of Physics Hubei Minzu University Enshi 445000 P. R. China; ^6^ Key Laboratory of Material Physics, Ministry of Education, School of Physics Zhengzhou University Zhengzhou 450001 P. R. China; ^7^ State Key Laboratory for Artificial Microstructure and Mesoscopic Physics and School of Physics Peking University Beijing 100871 P. R. China; ^8^ School of Electronics and Information Hangzhou Dianzi University Hangzhou 310018 P. R. China

**Keywords:** antiferromagnetic tunnel junction, Mn_3_SnN, SrTiO_3_, multiple resistance states, non‐collinear quantum transport simulations

## Abstract

Antiferromagnets have attracted widespread interest due to the advantages of no stray fields and ultrafast switching dynamics, promising for next‐generation high‐speed, high‐density memories. However, over a long period, the effective detection of antiferromagnetic (AFM) orders remained being one of the greatest challenges of its application in magnetic random access memories (MRAM) because of its zero net magnetization. Recently, the preliminary demonstration of the tunneling magnetoresistance ratio(TMR) in antiferromagnetic tunnel junctions (AFMTJ) offered a feasible solution. Here, a Mn_3_SnN/SrTiO_3_/Mn_3_SnN non‐collinear AFMTJ is designed and its transport properties are predicted by ab initio quantum transport simulations. Due to the momentum matching between the spin‐polarized Fermi surface of the Mn_3_SnN electrode and the low‐decay‐rate evanescent states of the SrTiO_3_ barrier, a remarkable TMR ≈1500% is generated, corresponding to a large device read margin, resulting in higher storage density. In addition, changing the relative orientation of two Mn_3_SnN magnetic orders leads to four non‐volatile resistance states with a low resistance area (RA) of only 0.07–1.25 Ω•µm^2^ and three multi‐state TMR of ≈500, 1000, and 1500%, suitable for high‐energy‐efficiency multiple‐state memory application. Our work provides a promising device structure for future nonvolatile high‐speed, high‐density, and multiple‐state AFM memories.

## Graphical abstract

A non‐collinear Mn_3_SnN/SrTiO_3_/Mn_3_SnN (001) AFMTJ is proposed with a theoretical TMR exceeding 1500%, enabled by optimal momentum (***k***)‐space matching between Mn_3_SnN's spin‐polarized Fermi surface ($p_{k_{\left|\right|}}$) and the low‐decay‐rate ($\kappa_{k_{\left|\right|}}$) of the SrTiO_3_ barrier. The eight degenerate AFM states of Mn_3_SnN enable multistate resistance and thus three distinct TMR values, offering a promising platform for future functional antiferromagnetic MRAM.

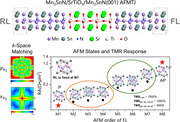

## 1 Introduction

As an emerging non‐volatile memory technology, magnetic random access memory (MRAM) is considered a competitive alternative for future standalone and embedded memory with key features and advantages, including non‐volatility, high speed, long‐term endurance, low‐power consumption, and radiation tolerance.^[^ ^1^, ^2^ ^]^ Magnetic tunnel junctions (MTJs) are the building blocks of MRAM.^[^ ^3^, ^4^, ^5^ ^]^ Current MTJ based on ferromagnetic materials suffers from data preservation difficulties and external magnetic field interference, limiting the further improvement of MRAM performance. Collinear antiferromagnetic materials, with zero stray field, high stability, and terahertz frequency, can provide better data stability, higher storage density, and higher writing speed, making them promising candidates for next‐generation memory technology.^[^ ^6^, ^7^, ^8^ ^]^ However, over a long period, antiferromagnetic materials are difficult to detect and manipulate because of their zero net magnetization.^[^ ^9^, ^10^ ^]^

Recent theoretical predictions of TMR in AFMTJs reveal a new mechanism for generating TMR, where the non‐relativistic momentum‐dependent spin polarization of the Fermi surface in both AFM electrodes plays a major role.^[^ ^11^, ^12^, ^13^ ^]^ However, for most AFM metals like *L*1_0_‐IrMn,^[^ ^14^ ^]^ CuMnAs,^[^ ^15^ ^]^ Mn_2_Au,^[^ ^16^, ^17^ ^]^ NiO,^[^ ^18^ ^]^ the spin degeneracy of their electronic band structures makes TMR unfeasible in the manner mentioned above.^[^ ^11^ ^]^ In recent years, the theoretical prediction and experimental observation of AFM spin polarization,^[^ ^19^, ^20^, ^21^, ^22^ ^]^ along with the development of spin group theory^[^ ^23^, ^24^, ^25^, ^26^, ^27^ ^]^ that neglecting spin‐orbital coupling (SOC), have provided a clear understanding of the symmetry requirements for AFM spin polarization. Generally, the conditions for AFM spin polarization are as follows: (1) the absence of the combination of space inversion and time reversal $\hat{P}\hat{T}$, and (2) the point group part of the spin translation group does not contain *D_n_*.^[^ ^25^ ^]^ When these conditions are satisfied, AFM spin polarization can typically be much stronger than the spin polarization generated by SOC, and thus result in significant TMR. For example, TMR of 500 and 300% in RuO_2_/TiO_2_/RuO_2_ (001) and Mn_3_Sn/Vacuum/Mn_3_Sn AFMTJs were theoretically predicted, respectively.^[^ ^11^, ^28^ ^]^ Experimentally, all‐antiferromagnetic tunneling junctions have only been demonstrated in non‐collinear antiferromagnetic junctions, Liu et al. have successfully fabricated MnPt/Mn_3_Pt/MgO/Mn_3_Pt AFMTJ and a TMR up to 100% has been observed at room temperature (300 K).^[^ ^29^ ^]^ Nakasuji et al. have fabricated Mn_3_Sn/MgO/Mn_3_Sn AFMTJ using molecular beam epitaxy and demonstrated a TMR of ≈2% at room temperature.^[^ ^30^ ^]^ These developments validate the feasibility of such AFMTJs, particularly demonstrating that non‐collinear antiferromagnets with weak net magnetic moments exhibit rich spin transport behaviors comparable to ferromagnets, positioning them as crucial components in future spintronic devices after ferromagnetic tunnel junctions.

**Figure 1:**
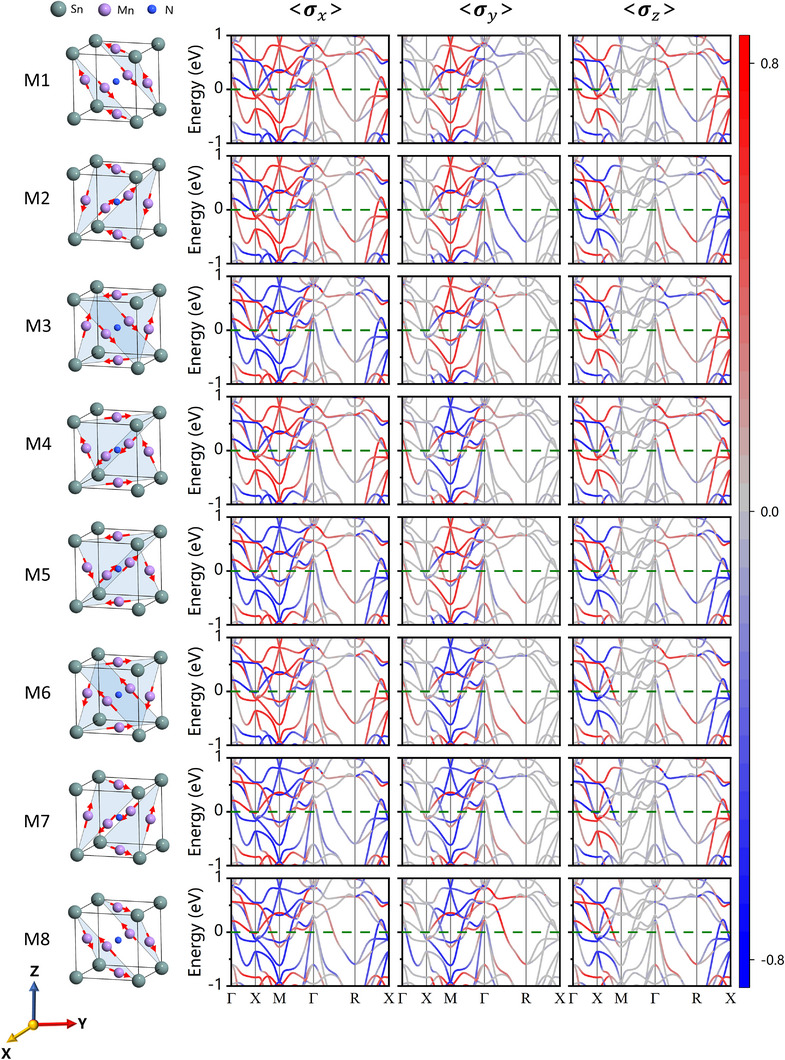
The geometric (left) and electronic structures (right) of Mn_3_SnN with eight symmetry‐equivalent ground AFM states (M1‐M8). The corresponding band structures along a high‐symmetry path in the Brillouin zone are shown with spin expectation values <σ_*x*_>, <σ_*y*_> and <σ_*z*_> represented by color. The color scale for the spin values is shown on the right.

Now that the TMR effect in AFMTJ has been verified, more effort should be put into exploring competitive AFM materials to generate a giant TMR favorable for practical applications. Crystallizing in the cubic lattice with $Pm\bar{3}m$ space group, Mn_3_SnN has the highest Néel temperature (T_N_ = 475 K) in the antiperovskite manganese nitride family,^[^ ^31^, ^32^, ^33^, ^34^ ^]^ surpassing Mn_3_GaN^[^ ^35^ ^]^ (T_N_ = 345 K) and Mn_3_NiN (T_N_ = 266 K).^[^ ^33^ ^]^ Besides, Mn_3_SnN exhibits excellent epitaxy with the commonly used MgO barrier and enables precise adjustment of epitaxial strain by fine‐tuning the nitrogen content, demonstrating its experimental and technological superiority. Below the Néel temperature, Mn_3_SnN exhibits a non‐collinear AFM order, where, within the two opposite planes perpendicular to the diagonal, Mn atoms form a Kagome‐type lattice with neighboring Mn magnetic moments aligned under 120° angle with respect to each other (**Figure** **1**).^[^ ^36^, ^37^ ^]^ Belonging to ‐ $R\bar{3}m$ ‐ magnetic space groups (MSGs),^[^ ^32^ ^]^ spin degeneracy of Mn_3_SnN electronic bands is broken, and momentum‐dependent spin splitting exists. Therefore, it's quite reasonable to deduce that the Mn_3_SnN‐based AFMTJ has non‐vanishing and even giant TMR.

In addition, Mn_3_SnN‐based AFMTJs hold promise for easier multistate differentiation once the large TMR is demonstrated. The study of multi‐state MTJs is valuable for increasing storage density and processing efficiency by expanding resistance states, overcoming binary storage limitations, and broadening application potential in neuromorphic computing.^[^ ^38^, ^39^, ^40^, ^41^, ^42^, ^43^ ^]^ Compared to methods like multilayer structures^[^ ^39^, ^44^ ^]^ or voltage control,^[^ ^41^, ^45^ ^]^ using non‐collinear AFM materials for multi‐state switching adds no structural complexity. It naturally enables multi‐state resistance through multiple symmetry‐equivalent AFM states, simplifying device design while retaining the aforementioned advantages of AFMTJs.^[^ ^11^ ^]^

In this work, by first principle ab initio quantum transport simulations,^[^ ^46^ ^]^ we predict that the Mn_3_SnN/SrTiO_3_/Mn_3_SnN non‐collinear AFMTJs exhibit giant TMR. We demonstrate that the non‐collinear AFM metal Mn_3_SnN hosts momentum‐dependent spin conduction channels controlled by its magnetic orders. Mn_3_SnN shows relatively high effective spin polarization in a cross‐section pattern of the Brillouin zone (BZ), matching well with the low‐decay‐rate evanescent states of the tunnel barrier SrTiO_3_. Thus, a giant TMR above 1500% is generated from the designed Mn_3_SnN/SrTiO_3_/Mn_3_SnN AFMTJ, indicating a large device read margin and a higher storage density. As bias voltage increases, TMR undergoes decreasing but can still keep ≈600% at 0.05 V, outperforming previous predictions of AFMTJ.^[^ ^11^, ^28^ ^]^ Remarkably, by changing the two Mn_3_SnN ground AFM orders, four groups of non‐volatile resistance states, together with low resistance area (RA) ranging from 0.07 to 1.25 Ω•µm^2^, are obtained, showing the promise for high density and high‐energy‐efficiency multiple‐state memory application.

## 2 Methodology

The geometry optimization and electronic structure computations mentioned in the manuscript are calculated using the density‐functional theory (DFT) implemented in the Vienna ab initio simulation package (VASP) code.^[^ ^47^, ^48^ ^]^ The electron‐ion interaction is described using the projected augmented plane‐wave (PAW) method^[^ ^49^, ^50^ ^]^ with a plane‐wave cut‐off energy set at 500 eV. The exchange‐correlation potential is characterized within the framework of generalized gradient approximation (GGA).^[^ ^51^ ^]^ Specifically, for the static electronic calculation of Mn_3_SnN, a *Γ*‐centered grid of 16×16×16 ***k***‐points is used to sample the Brillouin zone.^[^ ^52^ ^]^ The energy is converged to within 10^−5^ eV between two successive steps. For the Mn_3_SnN/SrTiO_3_/Mn_3_SnN AFMTJ, the interface between Mn_3_SnN and SrTiO_3_ is modeled by fixing the Mn_3_SnN lattice and stretching SrTiO_3_ (a = 3.945 Å), and the contact mismatch is only 1.67%. During the structure optimization, the nearest three layers of atoms on either side of the interface are allowed to relax and ensure the whole system can converge to a stable energy state. At least 20 Å vacuum buffer space is set along the contact directions to ensure decoupling between neighboring slabs. A *Γ*‐centered grid of 16×16×1 ***k***‐points is used to sample the Brillouin zone. The maximum force is less than 10^−2^ eV Å^−1^ per atom.

The transmission and complex band structure calculations of the AFMTJs are calculated by the DFT coupled with nonequilibrium Green's function (NEGF).^[^ ^46^, ^53^ ^]^ This solution has been effectively employed in previous studies to investigate the transport behavior of magnetic tunnel junctions.^[^ ^11^, ^28^, ^54^, ^55^ ^]^ The supercell Mn_3_SnN/SrTiO_3_/Mn_3_SnN is used as the scattering region, ideally attached to semi‐infinite Mn_3_SnN leads on both sides. The boundary conditions within the central zone are periodic along the *x* and *y* directions, and Dirichlet along the *z* direction. The calculation utilizes the non‐collinear GGA with PBE exchange‐correlation potential and employs a double zeta polarized (DZP) basis set.

The transmission coefficient $T_{\sigma }^{k_{\left|\right|}}\left(E\right)$ is calculated by:^[^ ^56^ ^]^

(1) $$T_{\sigma }^{k_{\left|\right|}}\left(E\right)=\mathrm{Tr}\left[n_{\sigma }\Gamma_{l,\sigma }^{k_{\left|\right|}}\left(E\right)G_{\sigma }^{k_{\left|\right|}}\left(E\right)\Gamma_{r,\sigma }^{k_{\left|\right|}}\left(E\right)G_{\sigma }^{k_{\left|\right|}†}\left(E\right)\right]$$

***k***_**||**_ is the reciprocal lattice vector along the surface‐parallel direction (orthogonal to the transmission direction) in the irreducible BZ. σ denotes the spin index, *n*_σ_ is the density matrix in the spin Hilbert space.$\;G_{\sigma }^{k_{\left|\right|}}\left(E\right)$ and $G_{\sigma }^{k_{\left|\right|}†}\left(E\right)$ represent the retard and advanced Green's function, respectively. $\Gamma_{l/r,\sigma }^{k_{\left|\right|}}\left(E\right)$ is the imaginary part of self‐energy indicating the coupling between the left/right electrode and the center region. To compute the transmission, the *k*‐point meshes for the electrode region and the central tunneling region are 13×13×100 and 13×13×1, respectively.

The transport current is calculated by the Landauer‐Bűttiker equation:^[^ ^57^ ^]^

(2) $$I_{\sigma }\left(V_{b}\right)=\frac{e}{h}\underset{-\infty }{\int^{+\infty }}\left\{T_{\sigma }\left(E,V_{b}\right)\left[\;f\left(E-\mu_{L}\right)-f\left(E-\mu_{R}\right)\right]\right\}\mathrm{dE}$$

where *T_σ_* (*E*, *V*_b_), *f*, *µ*_L/R_, and *T*(*E*) are the transmission coefficient, the Fermi‐Dirac distribution function, the Fermi level of the left/right electrode, and the averaged $T_{\sigma }^{k_{\left|\right|}}\left(E\right)$ over all different ***k***_**||**_, respectively.

## 3 Results

### 3.1 Electronic Structure of the Mn_3_SnN/SrTiO_3_ Interface

Our calculations correctly reproduce the electronic structure of bulk Mn_3_SnN, which is an itinerant magnetic metal with a non‐collinear antiferromagnetic ground state.^[^ ^32^, ^36^, ^58^ ^]^ The relaxed lattice parameter of Mn_3_SnN is 3.879 Å, and the magnetic moment per Mn atom is ≈2.5 µB. Below the Néel temperature, Mn_3_SnN displays a non‐collinear AFM arrangement, in which Mn atoms within the opposing planes that are perpendicular to the diagonal form a Kagome‐type lattice with adjacent Mn magnetic moments oriented at a 120° angle relative to each other.^[^ ^36^, ^37^ ^]^ This arrangement belongs to the magnetic space group $R\bar{3}m$. The geometric and electronic structures of Mn_3_SnN with eight symmetry‐equivalent ground AFM states (M1‐M8) are shown in Figure 1. The corresponding band structures along the high‐symmetry path of the Brillouin zone are also shown with spin expectation values <σ_*x*_>, <σ_*y*_> and <σ_*z*_> represented by color. Since the SOC effect is negligible on Mn_3_SnN's bandstructure (see Note S1, Supporting Information for more details), it is no longer considered in subsequent calculations for computational efficiency. Obviously, the spin component (<σ_*x*_>, <σ_*y*_> and <σ_*z*_>) in the energy band varies a lot between different AFM states. In a unit cell, when the magnetic moment of all Mn atoms flips 180 degrees (for example, M1 and M8), the contribution of a specific spin component is totally reversed.

**Figure 2:**
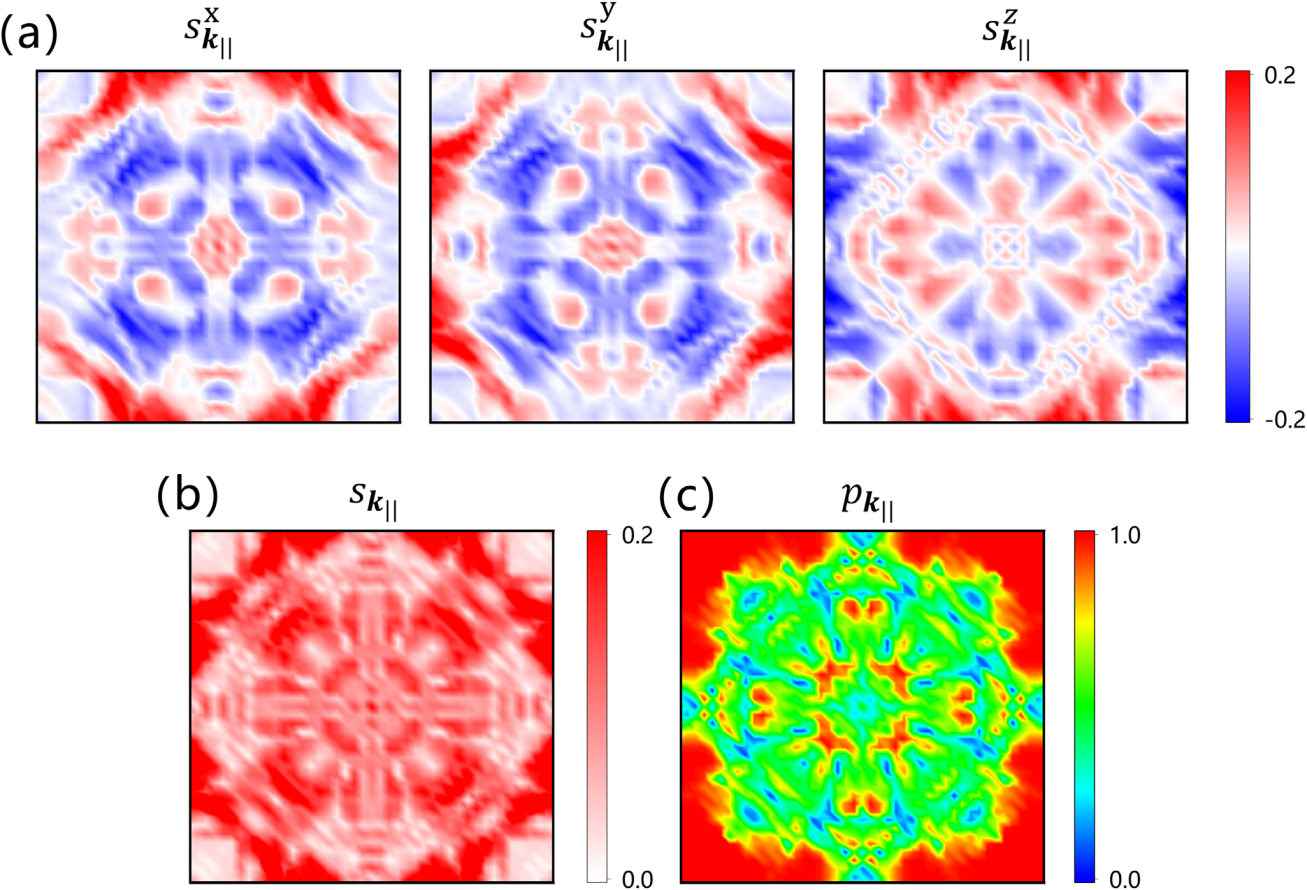
Components of the net spin $s_{k_{∥}}=\left(s_{k_{∥}}^{x},s_{k_{∥}}^{y},s_{k_{∥}}^{z}\right)$ a) and $s_{k_{∥}}$ ≡ |$s_{k_{∥}}$| b) in the 2DBZ of Mn_3_SnN (001) with AFM order M1. c) The magnitude of the effective spin polarization $p_{k_{∥}}$ = |$p_{k_{∥}}$| in the 2DBZ of Mn_3_SnN (001).

The non‐collinear AFM structure of Mn_3_SnN lifts the spin degeneracy, leading to the spin‐split Fermi surface holding conduction channels with a finite net spin $s_{k_{∥}}$.^[^ ^13^, ^14^ ^]^ Here, the net spin $s_{k_{∥}}$ is quantified in terms of the net spin $s_{k_{∥}}$ of the conduction channels available in electrodes at the transverse wave vector ***k***_**||**_:^[^ ^13^ ^]^

(3) $$s_{k_{∥}}=\sum_{n}s_{k_{∥}n}=\sum_{n}\frac{l_{z}}{2\pi }\int \left\langle nk\left|s\right|nk\right\rangle \delta \left(E_{nk}-E_{F}\right)dk_{z}$$

where 𝑙_z_ is the lattice constant of the electrode along the transport direction, 〈𝑛𝒌|𝒔|𝑛𝒌〉 is the spin expectation value for band 𝑛 with energy 𝐸*_n_*_𝒌_ at wave vector 𝒌 = (𝒌_**||**_, *k*_z_), and 𝐸_F_ is the Fermi energy. **Figure** **2**a,b show the calculated components of the net spin $s_{k_{∥}}=\left(s_{k_{∥}}^{x},s_{k_{∥}}^{y},s_{k_{∥}}^{z}\right)$ in the 2D Brillouin zone (2DBZ) of Mn_3_SnN(001) with AFM order M1. Due to a mirror plane ${\hat{M}}_{110}$ and a two‐fold rotation axis ${\hat{C}}_{110}$, $s_{k_{∥}}^{z}$ vanishes along the diagonals parallel to $k_{110}$ and $k_{\bar{1}10}$ in the 2DBZ. Once $s_{k_{∥}}$ is confirmed, the ***k***‐dependent spin polarization $p_{k_{∥}}$ at the Fermi energy can be generated from $\frac{s_{k∥}}{\sum_{n}\left|s_{k∥n}\right|}$, as shown in Figure 2c. It is notable that the high spin polarization is distributed around the central cross region (peaks) and the four corners (zones) of the 2DBZ.

**Figure 3:**
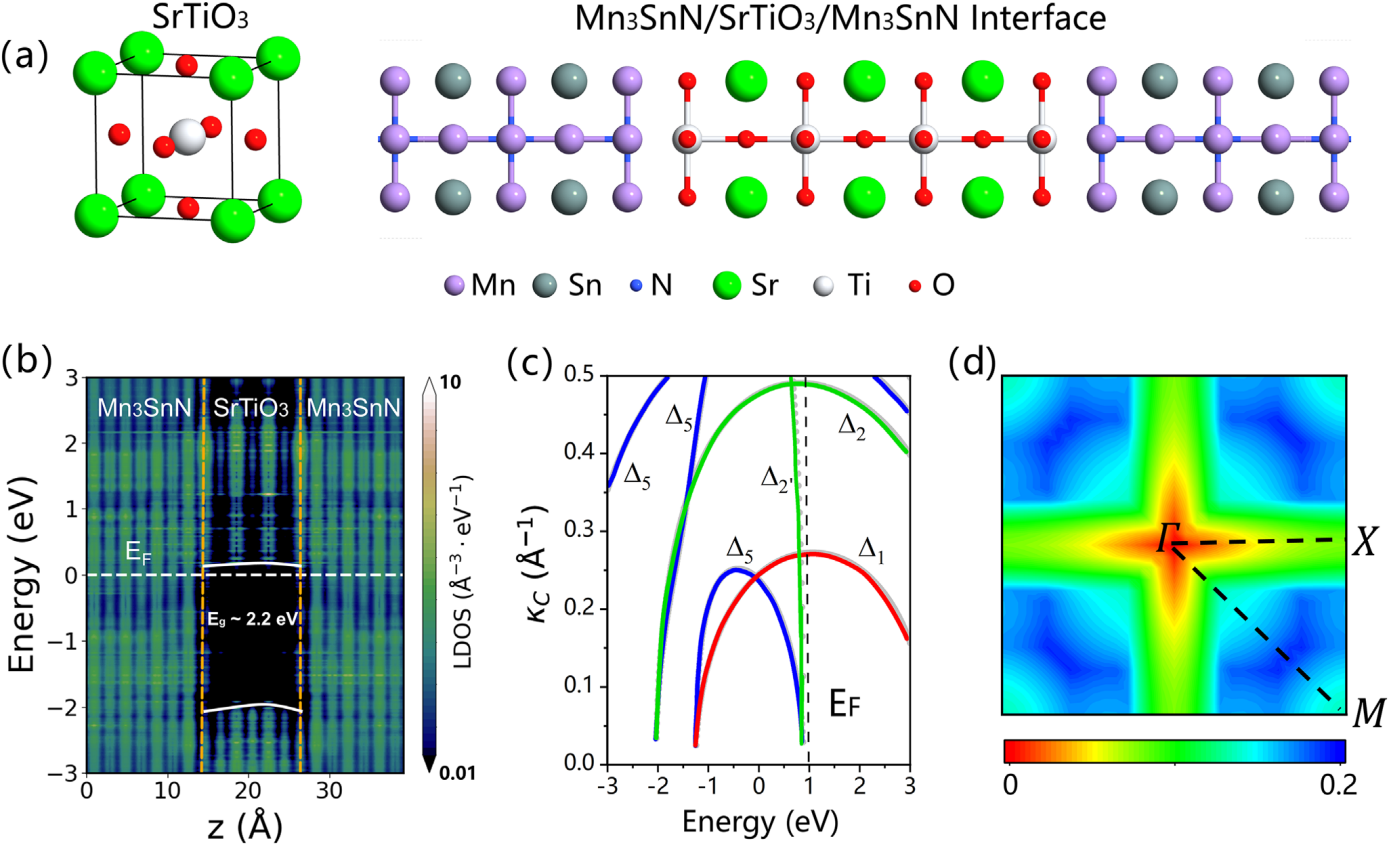
a) The atomic structure of SrTiO_3_ and the optimized interface of Mn_3_SnN/SrTiO_3_/ Mn_3_SnN. b) Projected local density of states (PLDOS) of Mn_3_SnN/SrTiO_3_/Mn_3_SnN AFMTJ. Yellow dashed lines: The interface between Mn_3_SnN and SrTiO_3_; White solid line: Band edge of the SrTiO_3_ barrier region. The Fermi level E_F_ is set at zero and denoted by the white dashed line. c) Complex band structures of SrTiO_3_ at the Γ (𝒌_**||**_ = 0) point. The position of the E_F_ in a Mn_3_SnN/SrTiO_3_/Mn_3_SnN is shown by the black dashed line. d) The lowest decay rate of the evanescent states of SrTiO_3_ (001) in the 2DBZ was calculated at the energy close to the bottom of the conduction band.

In order to design a Mn_3_SnN‐based AFMTJ in practice and guarantee a large TMR, two requirements need to be satisfied: (1) The barrier's lattice needs to match the magnetic electrode and form an energetically stable interface; (2) The ***k***‐resolved state of the barrier with the lowest decay rate should match the highly spin‐polarized Fermi surface of the electrode in momentum, thereby ensuring sufficient spin filtering effect.^[^ ^59^, ^60^ ^]^ SrTiO_3_ fulfills the above requirements quite well. First, the lattice parameter of SrTiO_3_ is 3.945 Å, very close to the 3.879 Å of Mn_3_SnN (the mismatch is only 1.67%), which allows the realization of high‐quality epitaxial heterostructures for device fabrication.^[^ ^35^, ^61^ ^]^ Different interfacial terminations are taken into consideration (see Note S2, Supporting Information for more details), and the most energy‐favorable Mn_3_SnN/SrTiO_3_ interface is made up of NMn_2_/TiO_2_ interface (**Figure** **3**a) with a distance of 2 Å. Second, the coincidence between the high‐$p_{k_{∥}}$ region (Figure 2c) and low‐decay‐rate area (Figure 3d) in the 2DBZ strongly indicates that Mn_3_SnN and SrTiO_3_ form a promising material pair for an AFMTJ capable of exhibiting giant TMR.

As is well known, the barrier's filtering efficiency can be reflected by the evanescent states from its complex band structure and Fermi surface *k*‐distribution. The symmetry analysis of evanescent states is of significant importance for understanding the tunneling mechanism. Here, the orbital components of each symmetry can be divided into Δ_1_ ($s,p_{z},d_{z^{2}}$) with spherical symmetry, Δ_5_ ($p_{x},p_{y},d_{\mathrm{xz}},d_{\mathrm{yz}}$) with twofold rotational symmetry, Δ_2_ ($d_{x^{2}-y^{2}}$) and Δ_2′_ ($d_{\mathrm{xy}}$) with other symmetries.^[^ ^62^, ^63^ ^]^ The projected local density of states (PLDOS) of the Mn_3_SnN/SrTiO_3_/Mn_3_SnN AFMTJ are shown in Figure 3b, where an *n*‐type Schottky contact (with barrier height of ≈0.15 eV) is generated at the interface; meanwhile, the SrTiO_3_ barrier region keeps an obvious band gap of 2.2 eV, which matches well with previous DFT calculation.^[^ ^64^ ^]^ Figure 3c exhibits the complex band structure of SrTiO_3_ at the Γ (𝒌_**||**_ = 0) point, in which the horizontal axis represents energy, while the vertical axis represents the decay rate (κ_*C*_) along <001> direction of the evanescent states. The larger the κ_*C*_, the faster the attenuation rate of the corresponding evanescent states ($∼\exp(-2\kappa_{C}d)$). Therefore, the states with smaller κ_*C*_ play a major role during the tunneling process. The valence band (VB) and conduction band (CB) of SrTiO_3_ exhibit different symmetries, where the VB closest to the Fermi level (0 eV) of SrTiO_3_ has Δ_1_ and Δ_5_ symmetries, while the CB closest to the Fermi level (0 eV) of SrTiO_3_ has Δ_2′_ and Δ_5_ symmetries.^[^ ^65^, ^66^ ^]^ It should be noted that the definitions of CB and VB here are based on the distribution of complex bands relative to zero energy when κ_*C*_ is small. Since the *E*_F_ of the Mn_3_SnN/SrTiO_3_ interface lies closer to the CB of SrTiO_3_, the electron barrier height with Δ_2′_ and Δ_5_ symmetries is lower than the hole barrier height with Δ_1_ and Δ_5_ symmetries, thereby leading to an electron tunneling mechanism. Thus, the states with Δ_2′_ and Δ_5_ symmetries of Mn_3_SnN tunnel more efficiently through the SrTiO_3_ barrier.

Although the analysis at the *Γ* point is informative, it is insufficient because conductance is not solely determined by this point. This can be understood from Figure 3d, which shows the lowest decay rates of the evanescent states in 2DBZ at the Fermi energy. The decay rates in the “cross” area along *Γ‐X and Γ‐M* paths are close to those at the *Γ* point, where the lowest decay rate occurs. As the barrier thickness increases, the states in this cross area are expected to dominate conductance. Such a low‐decay‐rate cross pattern of SrTiO_3_ matches well with the high‐$p_{k_{∥}}$ region of Mn_3_SnN in 2DBZ, satisfying the conditions mentioned above for generating a large TMR. Thus, a giant TMR of the Mn_3_SnN/SrTiO_3_/Mn_3_SnN AFMTJ is anticipated.

### 3.2 Multi‐State Resistance and Giant TMR in Mn_3_SnN/SrTiO_3_/Mn_3_SnN AFMTJ

**Figure 4:**
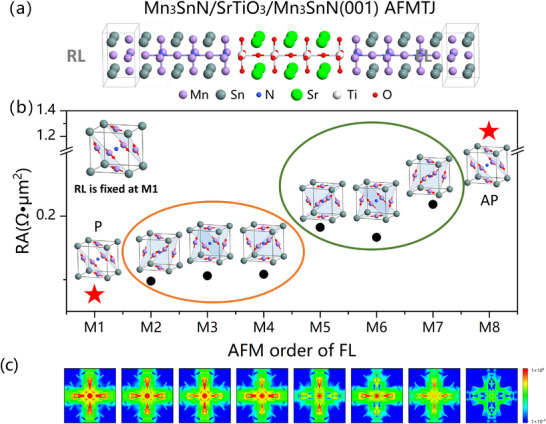
a) The atomic structure of Mn_3_SnN/SrTiO_3_/Mn_3_SnN (001) AFMTJ. The left Mn_3_SnN is treated as the reference layer (RL), while the right Mn_3_SnN is treated as the free layer (FL). b) Multi‐state resistance with AFMTJ's RL fixed at M1 AFM order. Four groups of resistance are obtained: M1(P) < M2, M3, M4 < M5, M6, M7 < M8(AP). P: parallel; AP: anti‐parallel. c) 2DBZ transmission spectrum of the AFMTJ when the FL adopts different AFM orders from M1 to M8 and the intensity is reflected by color.

**Figure** **4**a shows the perspective view of the atomic structure of Mn_3_SnN/SrTiO_3_/Mn_3_SnN (001) AFMTJ. Here, SrTiO_3_ with 7 atomic layers (7L) is adopted to generate symmetric interface contacts on both sides. The Mn_3_SnN layers on both sides of the AFMTJ adopt a semi‐infinite structure, with the left side as the reference layer (RL) and the right side as the free layer (FL). Here, we prefix M1 as the AFM order of the RL. By changing the AFM order of the FL from M1 to M8, the corresponding 8 resistance value of the AFMTJ can be calculated and categorized into four kinds of distinct resistance states, as shown in Figure 4b. Figure 4c illustrates the transmission behavior of the 2DBZ when the FL adopts different AFM orders from M1 to M8, thereby revealing the nature of resistance state changes. The overall cross‐shaped transmission spectrum of the 2DBZ reflects the filtering effect of SrTiO_3_. The detailed data can be found in **Table** **1**. Here are four categorized representative cases (see Note S3, Supporting Information for more details):

**Table 1:** Summary of the calculated transmission coefficient (*T*), resistance‐area (RA), and TMR of Mn_3_SnN/SrTiO_3_(7L)/Mn_3_SnN multi‐states AFMTJs at zero‐bias voltage. For the multi‐states AFMTJ, *TMR*_α − AP_ = (*T*_α_ − *T_AP_*)/*T_AP_* is adopted to describe the reading margin of the multi‐states at zero‐bias voltage.

AFM order of FL	AFM order of RL	*T*	RA (Ω•µm^2^)	*TMR*_α − AP_ (%)
M1 (P)	M1	5.09×10^−2^	0.076	1521
M2	M1	3.98×10^−2^	0.098	1168
M3	M1	3.66×10^−2^	0.106	1066
M4	M1	3.58×10^−2^	0.109	1040
M5	M1	2.13×10^−2^	0.182	578
M6	M1	2.33×10^−2^	0.167	642
M7	M1	1.78×10^−2^	0.218	467
M8 (AP)	M1	3.14×10^−3^	1.238	–

First, the FL of the AFMTJ is set under the M1‐type AFM order, where the $s_{k_{∥}}$($s_{k_{∥}}^{x},s_{k_{∥}}^{y},s_{k_{∥}}^{z}$) of FL and RL are identical. After the barrier's filtering, the remaining highly spin‐polarized transmission channels in the cross‐section of 2DBZ of FL and RL can be preserved maximally, resulting in the transmission coefficient being as high as 5.09×10^−2^ (corresponding to a small RA of 0.076 Ω•µm^2^).

Second, the AFM order of the FL is either under M2, M3, or M4 states, where one of the three spin components is reversed compared to M1. Take the FL under the M2 state as an example; its band structures with <σ_*z*_> reverse while <σ_*x*_> and <σ_*y*_> preserve compared to those of M1. Thus, the corresponding reversed $s_{k_{∥}}^{z}$ between the FL and RL leads to a reduced transmission coefficient of 3.98×10^−2^ (corresponding to RA of 0.098 Ω•µm^2^).

Third, the AFM order of the FL is either M5, M6, or M7 states, where two of the three spin components are reversed compared to M1. For instance, the $s_{k_{∥}}^{x}$ and $s_{k_{∥}}^{y}$ between the FL and RL is reversed when FL takes AFM order M7, leading to a further reduced transmission coefficient of 1.78×10^−2^ (corresponding to RA of 0.218 Ω•µm^2^).

Fourth, the AFM order of the FL is set to M8 states. Due to the completely reversed $s_{k_{∥}}$($s_{k_{∥}}^{x},s_{k_{∥}}^{y},s_{k_{∥}}^{z}$) in FL and RL, the ***k***‐resolved highly spin‐polarized transmission channels are totally suppressed, resulting in minimum transmission of only 3.14×10^−3^ (corresponding to RA of 1.238 Ω•µm^2^).

*TMR*_α − AP_ = (*T*_α_ − *T_AP_*)/*T_AP_* is adopted to describe the reading margin of the multi‐states AFMTJ at zero‐bias voltage, where *T*_α_ represents the device transmission coefficient in α state. Here, RL's AFM order is fixed at M1. When FL is in AFM order M1, the AFMTJ is in the P (parallel) state. When FL is in AFM order M8, the AFMTJ is in the AP (anti‐parallel) state. When FL is in AFM order M2 ‐ M7, the AFMTJ is in the intermediate state. The calculated maximum *TMR*_P − AP_ of the 7L barrier AFMTJ reaches as high as 1521%. The value of *TMR*_M2 − AP_, *TMR*_M3 − AP,_ and *TMR*_M4 − AP_ is ≈1000%. The value of *TMR*_M5 − AP_, *TMR*_M6 − AP_, and *TMR*_M7 − AP_ is ≈500%. Notably, the distinguishable TMR intervals between these multi‐states are ≈500%, about one‐third of the maximum TMR (1521%). This is mainly because the TMR magnitude of the AFMTJ is determined by the spin polarization matching between the FL and RL. In a word, the combinations of the eight symmetry equivalent ground AFM states result in four groups of distinctly distinguishable resistance states, which ultimately translate into three clearly identifiable TMR signals in the AFMTJ. Therefore, our theoretical calculations demonstrate that multi‐state storage is feasible for the designed Mn_3_SnN‐based AFMTJ, and these resistance states are distinguishable with relatively high TMR signals.

### 3.3 Barrier Layer‐ and Bias‐Dependent TMR of Mn_3_SnN/SrTiO_3_/Mn_3_SnN AFMTJ

**Figure 5:**
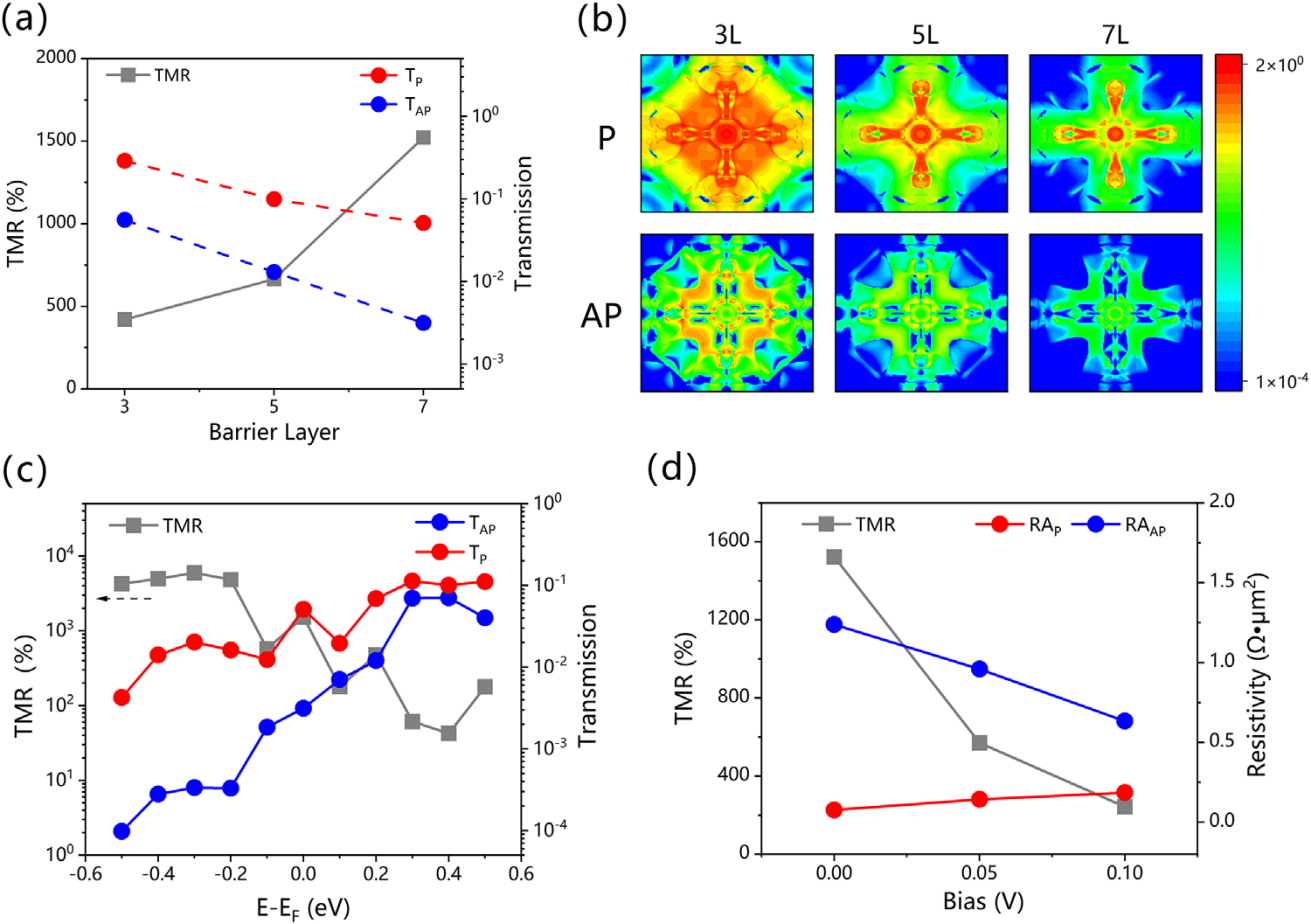
a) Total transmission at E_F_ and TMR as functions of barrier thickness. b) The calculated ***k***_**||**_‐resolved transmission in the 2DBZ for the AFMTJ with barrier thicknesses of 3L, 5L, and 7L. c) Total transmission and TMR as functions of energy. d) Resistivity and TMR as functions of bias voltage.

The effect of barrier layer thickness on the TMR and RA of the designed AFMTJ is also investigated, where SrTiO_3_ with 3 atomic layers (3L) and 5 atomic layers (5L) are taken into consideration. The barrier‐layer‐dependent transmission at *E*_F_ and TMR are shown in **Figure** **5**a, and the detailed data can be found in **Table** **2**. Figure 5b exhibits the transmission distribution in 2DBZ for AFMTJ with different barrier thicknesses. Clearly, as the thickness of the SrTiO_3_ increases from 3L to 7L, the enhanced tunneling distance strengthens the barrier filtering effect, reflected by the gradually apparent cross‐shaped transmission distribution (matching the aforementioned low‐decay‐rate evanescent states distribution of SrTiO_3_) and the overall decrease of T_P_ and T_AP_. More specifically, T_AP_ decreases faster than T_P_, resulting in an increase in TMR from 420% for 3L to 664% for 5L, and finally to 1521% for 7L. Although thickening the barrier layer brings about a large TMR due to the enhancement of filtering, it also causes a larger RA, which is detrimental to device miniaturization and chip storage density. According to the study of Yakushiji,^[^ ^67^ ^]^ for a 5 Gbit in^−2^ high‐density MRAM, the MTJ's RA product must be kept below 6 Ω•µm^2^. From Figure 5a and Table 2, we note that with the barrier layer increasing from 3L to 5L and finally to 7L, the maximal RA at AP state rises from 6.99×10^−2^ to 3.00×10^−1^ and finally to 1.24 Ω•µm^2^. Considering that the RA of the studied AFMTJ reaches the order of Ω•µm^2^ when the SrTiO_3_ is 7L thick, and given that inevitable defects and disorders introduced during actual experimental preparation will further increase the RA, exploring the performance with thicker barrier layers is of limited significance. From a theoretical calculation perspective, the Mn_3_SnN‐based AFMTJ with a 7L thick SrTiO_3_ exhibits a good balance between TMR and RA, achieving optimal performance.

**Table 2:** Summary of the calculated transmission coefficient (*T*), resistance‐area (RA), and TMR of Mn_3_SnN/SrTiO_3_/Mn_3_SnN AFMTJs without bias. TMR represents the TMR between P and AP states.

Tunneling barrier thickness		P	AP
3L	*T*	2.89×10^−1^	5.56×10^−2^
	RA (Ω•µm^2^)	1.34×10^−2^	6.99×10^−2^
	TMR (%)	420	
5L	*T*	9.90×10^−2^	1.30×10^−2^
	RA (Ω•µm^2^)	3.92×10^−2^	3.00×10^−1^
	TMR (%)	664	
7L	*T*	5.09×10^−2^	3.14×10^−3^
	RA (Ω•µm^2^)	7.63×10^−2^	1.24×10^0^
	TMR (%)	1521	

It's important to emphasize that the zero‐bias tunneling data and TMR analysis mentioned above are discussed only at the Fermi level, which is applicable under low‐temperature circumstances. In fact, the MTJ usually operates within the temperature window of − 40 − 120°C. As a result of thermal fluctuations, electrons in the vicinity of the Fermi surface contribute to the conductivity as well. Additionally, the system's Fermi level may shift due to oxidation and doping during the fabrication process. Therefore, investigating the tunneling at different energies is necessary (Figure 5c) for the guidance of the actual device operation and the lightly doped circumstance. Within the energy range of ±0.5 eV around the Fermi level, we found that the tunneling coefficients for the P and AP states of the system both exhibit an oscillating increase tendency with the increasing energy. Compared to the P state, the tunneling of the AP state rises more rapidly, leading to an overall decrease in TMR with increasing energy. At 0.2 eV above the Fermi energy, the tunneling probabilities of P state and AP state are comparable. At energies levels 0.2 eV below the Fermi energy, the TMR remains between 1000% and 10000%. This phenomenon can be explained easily. According to the band structure calculation, the *E*_F_ of the interfacial system is close to the CBM of SrTiO_3_. If the *E*_F_ is raised (like electron doping) above the CBM, electrons injected from the Mn_3_SnN electrode to the SrTiO_3_ barrier do not need to pass through any contact barrier. In this case, the electron will directly pass rather than tunnel through the barrier, weakening the filtering effect of the barrier to a certain degree and naturally leading to a decrease in TMR. If the *E*_F_ is lowered (like hole doping) within the middle of SrTiO_3_, the interface barrier will hinder the electrons from migrating from the Mn_3_SnN electrode to the SrTiO_3_ barrier. Therefore, electrons tend to reach the opposite electrode through tunneling, and the filtering effect of the barrier layer will be fully utilized, resulting in an increase in TMR. Thus, it can be reasonably inferred that a device with light hole doping is expected to achieve a higher TMR.

Reading operation of MRAM requires applying a small bias voltage across the MTJ to determine the resistance state based on the magnitude of the current signal. Under different biases, the chemical potential of the magnetic electrodes on both sides changes, altering the transmission energy window and thus affecting TMR. To evaluate the effect of the applied bias, we calculate the IV outputs under the P and the AP states and generate the corresponding TMR, as shown in Figure 5d. When *V*_b_ is applied, the TMR definition becomes:

(4) $$\mathrm{TMR}\;=\frac{I_{P}-I_{\mathrm{AP}}}{I_{\mathrm{AP}}}\times 100\%$$

We study the performance of TMR with bias voltages of 0.05 and 0.1 V, shown in Figure 5d and **Table** **3**. As the bias voltage increases, the mismatch between the chemical potentials of the Mn_3_SnN electrodes on both sides increases. Considering the P state, the matching of ***k***‐resolved high‐spin‐polarization channels between the two Mn_3_SnN electrodes decreases with increasing bias voltage, leading to a decrease in the transmission probability, reflected by an enlarged RA_P_. In the AP state, however, the electrodes on both sides cannot fully exhibit the reversed spin polarization $s_{k_{∥}}$($s_{k_{∥}}^{x},s_{k_{∥}}^{y},s_{k_{∥}}^{z}$) due to the chemical potentials mismatch. As a result, the transmission of AP state can't be surpassed effectively but shows an increasing trend, reflected by a reduced RA_AP_. Overall, the device's TMRs decrease with increasing bias voltage (Figure 5d). However, it's worth noting that even under the typical bias voltage of 0.05 V, the TMR of the studied AFMTJs with different barrier thicknesses still remains between 200% and 600%, meeting the practical criteria.

**Table 3:** Summary of the calculated transport properties at Fermi level, and TMR of Mn_3_SnN/SrTiO_3_/Mn_3_SnN AFMTJs with bias.

Tunnel barrier thickness	Bias (*V*)	*I*_P_ (*A*)	RA_P_ (Ω•µm^2^)	*I*_AP_ (*A*)	RA_AP_ (Ω•µm^2^)	TMR (%)
3L	0.05	4.16×10^−7^	0.018	1.25×10^−7^	0.060	234
	0.1	1.05×10^−6^	0.014	5.79×10^−7^	0.026	81
5L	0.05	1.17×10^−7^	0.064	3.22×10^−8^	0.234	265
	0.1	1.72×10^−7^	0.087	6.58×10^−8^	0.229	162
7L	0.05	5.27×10^−8^	0.143	7.84×10^−9^	0.959	572
	0.1	8.14×10^−8^	0.185	2.38×10^−8^	0.633	242

## 4 Discussion

### 4.1 Barrier Material Considerations Beyond SrTiO_3_

To assess the generality of our findings, we further investigated alternative oxide barriers, including BaTiO_3_, CaTiO_3_, and MgO. BaTiO_3_ and CaTiO_3_ share the perovskite structure with SrTiO_3_ and exhibit acceptable lattice mismatches with Mn_3_SnN (2.88% and 0.27%, respectively). BaTiO_3_ has a smaller bandgap (≈1.8 eV), which limits its tunneling selectivity, and its non‐ideal ***k***‐space matching with the high‐$p_{k_{∥}}$_*‐*_ Fermi surface of Mn_3_SnN results in a low TMR (≈37%). CaTiO_3_, despite having a comparable bandgap to SrTiO_3_ (≈2.2 eV), exhibits less favorable momentum alignment and thus yields a reduced TMR (≈200%). SrTiO_3_, by contrast, provides both effective filtering and good ***k***‐space matching, leading to a much higher TMR (≈1500%). Additionally, we examined MgO—a commonly used barrier in conventional MTJs—by employing a strained interface model to accommodate its large lattice mismatch (≈7.89%) with Mn_3_SnN. While this model may not fully capture realistic interfacial behavior, the result indicates that MgO could achieve a high TMR (≈5000%) by leveraging its large bandgap (≈5.0 eV) and favorable ***k***‐space tunneling characteristics near the *Γ*‐point. These results highlight the importance of both interfacial and electronic compatibility in AFMTJ barrier selection (see Note S4, Supporting Information for more details).

### 4.2 Perspectives of Experimental Realization

The proposed AFMTJ is experimentally feasible based on our theoretical simulations. First, the fabrication of Mn_3_SnN films follows established protocols, with its anti‐perovskite structure in a cubic crystal system enabling high‐quality growth through sputtering on (001)/(111) SrTiO_3_ or MgO single‐crystal substrates, resulting in oriented epitaxial growth.^[^ ^32^, ^68^, ^69^ ^]^ Various cubic oxide systems, particularly SrTiO_3_ as confirmed by our calculations, can induce significant TMR and serve effectively as barrier layers for Mn_3_SnN AFMTJs. However, interfacial defects and lattice mismatches can significantly degrade TMR performance (See Note S5, Supporting Information for a detailed discussion). The lattice and transmission compatibility of Mn_3_SnN with various oxides facilitates the design of the film structures for Mn_3_SnN AFMTJs. Based on high quality film growth, several recent reports have highlighted TMR in AFMTJs composed of non‐collinear AFM Mn_3_Pt and Mn_3_Sn, especially in the AFMTJ with Mn_3_Pt, which exhibits room‐temperature TMR exceeding 100%.^[^ ^29^, ^30^, ^70^ ^]^

On the other hand, antiferromagnetic tunneling junctions require viable writing mechanisms, notably spin‐orbit torque. Experimentally, non‐collinear antiferromagnets can be characterized as the ferroic ordering structure of a cluster magnetic octupole, comprising six Mn atoms arranged in two triangular spin configurations.^[^ ^68^ ^]^ In the case of Mn_3_Sn, by precisely controlling its crystal orientation through epitaxial growth, a perpendicular cluster magnetic octopole easy axis is formed, thus realizing a 180° cluster magnetic octopole switching like a perpendicular ferromagnetic layer.^[^ ^71^ ^]^ On the other hand, for tilted cluster magnetic octopole, such as polycrystalline Mn_3_Sn, 180° field‐free switching of cluster magnetic octopole can be realized by combining in‐plane and out‐of‐plane SOTs generated by low‐symmetry spin sources.^[^ ^72^ ^]^ We highlight the advantages of Mn_3_SnN over Mn_3_Sn from a theoretical and experimental perspective (see Note S6, Supporting Information for details). Mn_3_SnN's analogous cluster magnetic octopole moment facilitates a viable protocol for the experimental realization of “parallel” and “antiparallel” states via cluster magnetic octopole moment switching through magnetic field or spin‐orbit torque.^[^ ^71^, ^72^ ^]^

Experimentally, in principle, Mn_3_SnN has six stable magnetic octopole states: *θ* = ±150°, ±30°, and ±90°, where *θ* is defined as the azimuthal angle of the magnetic octopole moment in the (111) kagome plane (see Note S7, Supporting Information for schematic diagram). Taking *θ* = +90° as the initial state, *θ* = +30° and +150° correspond to a rotation of 60°, *θ* = ‐30° and ‐150° correspond to a rotation of 120°, and *θ* = ‐90° corresponds to a rotation of 180°. The three rotation angles coincide with the three TMR values predicted by our theory. Reversible electrical manipulation of the magnetic octopole between the two closest energy minimum states *θ* = ±150° or ±30° can be achieved using the in‐plane spin *σ*_y_ generated by the spin Hall effect.^[^ ^73^, ^74^ ^]^ Heavy metals Pt or W are a promising candidate due to their large spin Hall angle. From an industrial perspective, Pt(W)/Mn_3_SnN/SrTiO_3_/Mn_3_SnN‐based antiferromagnetic tunnel junctions face the challenge of field‐free manipulation of cluster magnetic octopole moment, which could be addressed through precisely tuning the Mn_3_SnN symmetry or using low‐symmetry spin source. For example, field‐free 180° (*θ* = ±90°) electrical manipulation of the magnetic octupole can be achieved by utilizing a low‐symmetry spin source with *σ*_y_ and out‐of‐plane spin *σ*_z_.^[^ ^72^ ^]^

Furthermore, the on‐chip integration of these AFMTJ devices, including those based on Mn_3_SnN, presents challenges to current techniques. Recently, Parkin *et al.* demonstrated magnetic tunnel junction memory devices utilizing low‐magnetization ferrimagnetic Heusler alloy Mn_3_Ge as the storage layer on amorphous substrates, achieving 87% TMR through a combined nitride seed and chemical template layer approach.^[^ ^75^ ^]^ Similarly, in Si substrate/MgO seed layer/Mn_3_Sn heterostructures, post‐annealing techniques have yielded polycrystalline Mn_3_Sn with predominant [20$\bar{2}$1] crystal orientation.^[^ ^76^ ^]^ Additionally, all‐antiferromagnetic tunnel junctions on silicon substrates have reached 110% TMR using a Si/Pt/Mn_3_Pt (polycrystalline, predominantly (111)‐oriented)/Al_2_O_3_/Mn_3_Pt stack structure.^[^ ^70^ ^]^ These findings suggest the feasibility of producing selectively oriented Mn_3_SnN on silicon by optimizing seed or chemical template layers. Nevertheless, the practical implementation of antiferromagnets as information storage layers remains a long‐term research objective within antiferromagnetic spintronics. Our initial experiments have produced high‐quality Mn_3_SnN AFMTJ thin film structures on both single‐crystal and amorphous substrates, bolstering our confidence in the experimental verification of Mn_3_SnN AFMTJ.

## 5 Conclusion

To summarize, we devised a non‐collinear AFMTJ structure comprising Mn_3_SnN/SrTiO_3_/Mn_3_SnN and forecasted its transport characteristics using ab initio quantum transport simulations. Due to the momentum matching between the Fermi surface spin polarization of the Mn_3_SnN electrode and the low‐decay‐rate evanescent states of the SrTiO_3_ barrier, an impressive TMR exceeding 1000% is achieved. This could translate to a substantial device read margin and consequently enable higher storage density. Moreover, altering the AFM order of the two Mn_3_SnN electrodes results in four groups of non‐volatile resistance states characterized by a minimal resistance area (RA) of only 0.07–1.25 Ω•µm^2^, and three multi‐state TMR of ≈500, 1000, and 1500%, making them suitable for high‐energy‐efficient multiple‐state memory applications. Our study offers a promising material framework for forthcoming nonvolatile AFM memories characterized by high speed, high density, and multiple‐state capabilities.

## Conflict of Interest

The authors declare no conflict of interest.

## Supporting information



Supporting Information

## Data Availability

The data that support the findings of this study are available from the corresponding author upon reasonable request.
