# Supplementary material for: Mn3SnN‐Based Antiferromagnetic Tunnel Junction with Giant Tunneling Magnetoresistance and Multi‐States: Design and Theoretical Validation
Source: Adv Sci (Weinh). 2025 Jun 10;12(32):e02985. doi: 10.1002/advs.202502985 (PMC12407296; doi:10.1002/advs.202502985)
Supplement: Supplementary file 1 — Supporting Information [file ADVS-12-e02985-s001.docx]

**Supporting Information**

**Mn_3_SnN-based Antiferromagnetic Tunnel Junction with Giant Tunneling Magnetoresistance and Multi-states: Design and Theoretical Validation**

Shiqi Liu,^1, †^ Tingwei Chen,^1, †^ Baochun Wu,^2. †^ Haodong Fan,^1^ Yingmei Zhu,^1^ Sheng Bi,^3^ Yuntian Liu,^4^ Yinuo Shi,^1^ Wenbiao Zhang,^1^ Mengxi Wang,^1^ Qiang Li,^5^ Jie Yang,^6, *^ Jing Lu,^7^ Tiejun Zhou^1,8*^ and Bo Liu^1,*^

^1^ State Key Laboratory of Spintronics Devices and Technologies, Hangzhou 311305, P. R. China

^2^ State Key Laboratory of Low Dimensional Quantum Physics and Department of Physics, Tsinghua University, Beijing 100084, P. R. China

^3^ Research Center for New Materials Computing, Zhejiang Lab, Hangzhou 311100, P. R. China

^4^ Department of Physics and Shenzhen Institute for Quantum Science and Engineering (SIQSE), Southern University of Science and Technology, Shenzhen 518055, China

^5^ Department of Physics, Hubei Minzu University, Enshi, 445000, P. R. China

^6^ Key Laboratory of Material Physics, Ministry of Education, School of Physics, Zhengzhou University, Zhengzhou 450001, P. R. China

^7^ State Key Laboratory for Artificial Microstructure and Mesoscopic Physics and School of Physics, Peking University, Beijing 100871, P. R. China

^8^ School of Electronics and Information, Hangzhou Dianzi University, Hangzhou 310018, P. R. China

^†^These authors contributed equally to this work.

Email: yangjie_phy@zzu.edu.cn, [zhoutiejun@spinlab.cn](mailto:zhoutiejun@spinlab.cn), [liubo@spinlab.cn](mailto:liubo@spinlab.cn)

**Note 1. SOC Effect on Band Structures of Mn_3_SnN**

**
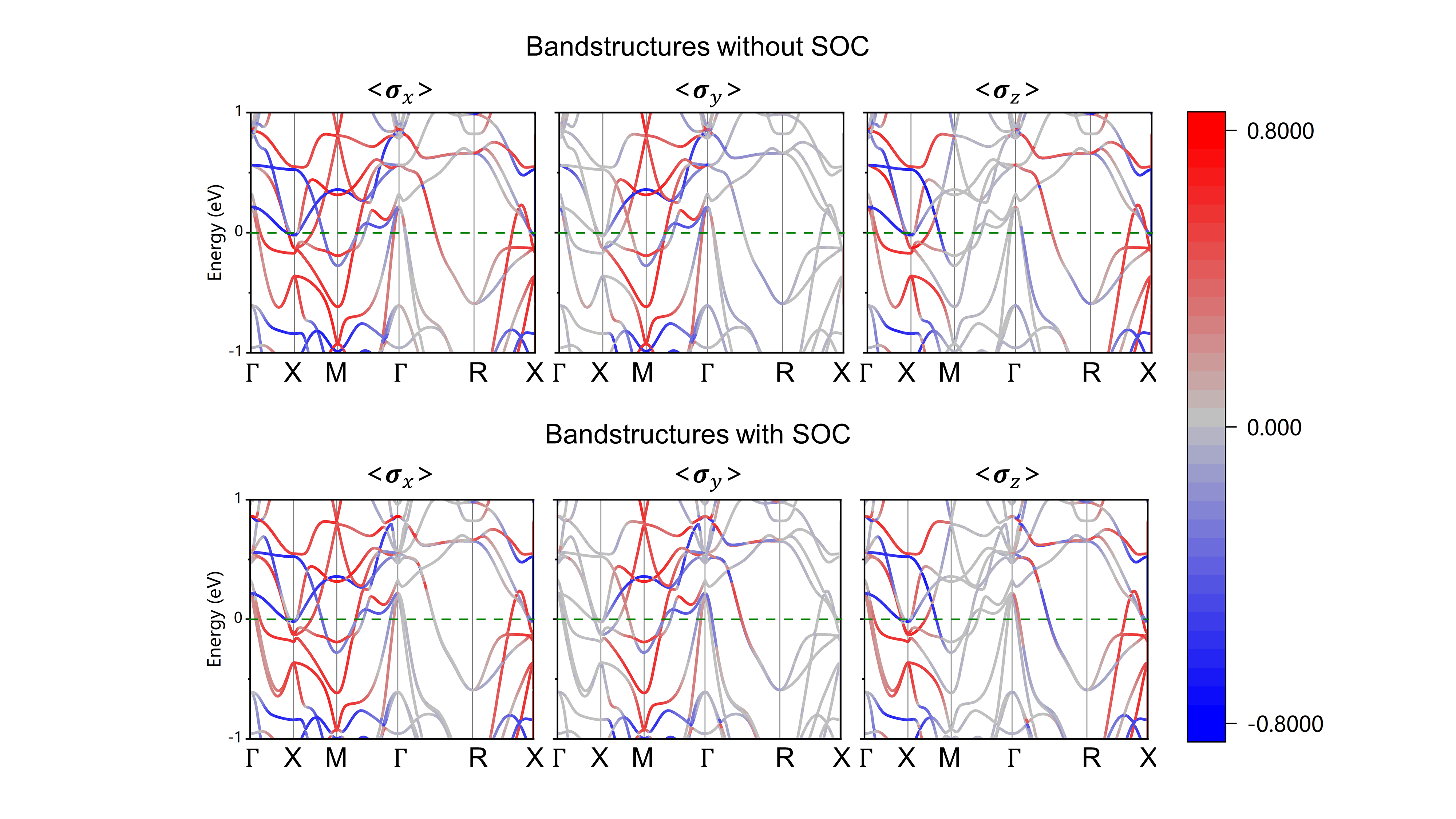
**

**Figure S1.** Band structures of Mn_3_SnN (M1 AFM order) without SOC (up panel) and with SOC (down panel). The corresponding band structures along the high-symmetry path in the Brillouin zone are shown with spin expectation values <$\sigma_{x}$>, <$\sigma_{y}$>, and <$\sigma_{z}$> represented by color. The color scale for the spin values is shown on the right.

Here, take the Mn_3_SnN with M1 AFM order as an example. Considering the SOC effect, only a tiny fraction of the energy band in the deep energy levels split, while the energy bands and spin expectation values near the Fermi energy change little. Since the electronic conduction is mainly concentrated near the Fermi energy, it can be assumed that the effect of SOC on the transport is negligible. Considering that the calculation of SOC is time-consuming, it is reasonable to ignore the SOC in subsequent calculations to ensure calculation efficiency and data reliability at the same time.

**Note 2. Tested Mn_3_SnN/SrTiO_3_ (001) Interface Configurations Stability**


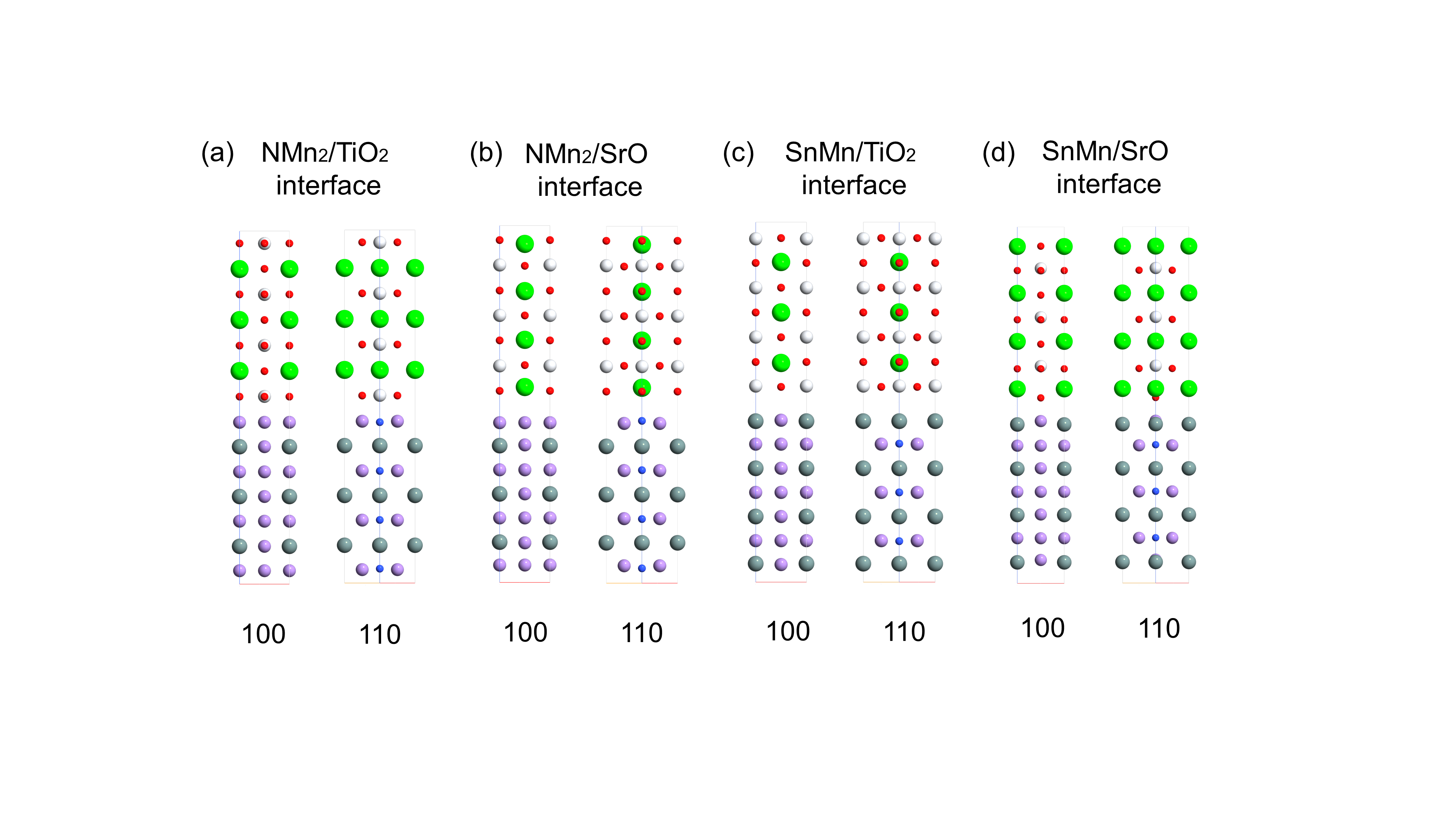


**Figure S2.** (a)–(d) Tested Mn_3_SnN/SrTiO_3_ (001) interface configurations with the view from <100> and <110> directions: NMn_2_/TiO_2_ (a), NMn_2_/SrO (b), SnMn/TiO_2_ (c), and SnMn/SrO (d).

The interface stability of the Mn_3_SnN/SrTiO_3_ (001) heterostructure was analyzed based on binding energy. The simulated four possible interfacial atomic configurations between Mn_3_SnN and SrTiO_3_ are shown in Fig.S2, with NMn_2_/TiO_2_, NMn_2_/SrO, SnMn/TiO_2_, and SnMn/SrO interface, respectively. Binding energy *E*_b_ is the energy per $Å^{2}$ required to remove SrTiO_3_ from the Mn_3_SnN surface and is calculated as:

$$E_{b}=\left( E_{\mathrm{Mn}_{3}\mathrm{SnN}/\mathrm{SrTi}O_{3}}-E_{\mathrm{Mn}_{3}\mathrm{SnN}}-E_{\mathrm{SrTi}O_{3}} \right)/A$$

Where $E_{\mathrm{Mn}_{3}\mathrm{SnN}}$, $E_{\mathrm{SrTi}O_{3}}$and $E_{\mathrm{Mn}_{3}\mathrm{SnN}/\mathrm{SrTi}O_{3}}$ represents the total energy of the pristine Mn_3_SnN, SrTiO_3_ and the interface system, respectively, and *A* is the area of the contact interface in the system. The lower the $E_{b}$ is, the more stable the configuration is.

**Table S1. Formation energies** $\boldsymbol{\Delta}\boldsymbol{E}$ **(eV/**$\boldsymbol{Å}^{\boldsymbol{2}}$**) for different interfaces in the Mn_3_SnN/SrTiO_3_ (001) heterostructure.**

| Interface Configuration | NMn_2_/TiO_2_ | NMn_2_/SrO | SnMn/TiO_2_ | SnMn/SrO |
| --- | --- | --- | --- | --- |
| $\Delta E$ | -3.43 | -2.25 | -2.22 | -1.45 |

As is evident from Table S1, all the interfaces have negative formation energies, which implies that they are energetically stable. There is strong binding at the NMn_2_/TiO_2_ interface with an apparent lower binding energy *E*_b_ of 3.43 eV/$\boldsymbol{Å}^{\boldsymbol{2}}$ than the formation energies of the other interface terminations. Thus, the most energy-favorable configuration has the NMn_2_/TiO_2_ interface, which is clean and sharp interface with a distance of 2Å.

**Note 3. Net spin** $\boldsymbol{s}_{\boldsymbol{k}_{\boldsymbol{\parallel}}}$**(**$\boldsymbol{s}_{\boldsymbol{k}_{\boldsymbol{\parallel}}}^{\boldsymbol{x}}\boldsymbol{,}\boldsymbol{s}_{\boldsymbol{k}_{\boldsymbol{\parallel}}}^{\boldsymbol{y}}\boldsymbol{,}\boldsymbol{s}_{\boldsymbol{k}_{\boldsymbol{\parallel}}}^{\boldsymbol{z}}$**) in the 2DBZ of Mn_3_SnN (001) with AFM order of M1, M2, M7, and M8**


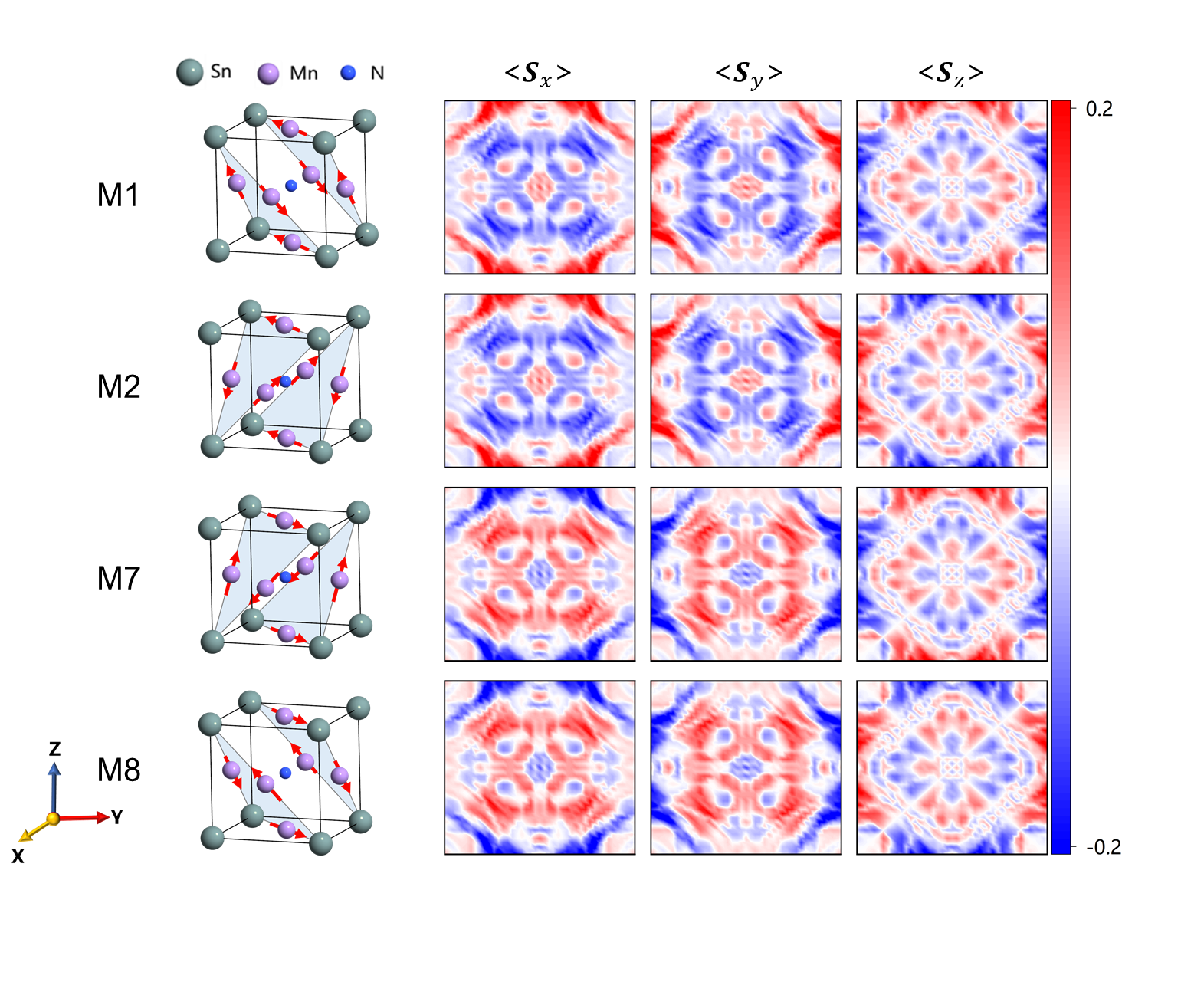


**Figure S3.** The atomic and magnetic structure of Mn_3_SnN in the non-collinear AFM order of M1, M2, M7, and M8, respectively. The corresponding net spin $\boldsymbol{s}_{\boldsymbol{k}_{\parallel}}$($s_{\boldsymbol{k}_{\parallel}}^{x},s_{\boldsymbol{k}_{\parallel}}^{y},s_{\boldsymbol{k}_{\parallel}}^{z}$) in the 2DBZ of Mn_3_SnN (001) indicated by color. The color scale for the spin values is shown on the right.

**Note 4. Potential Alternatives Barrier Materials Beyond SrTiO_3_**

We have systematically examined several potential alternatives, including BaTiO_3_ and CaTiO_3_ (both perovskites), as well as MgO, a widely used barrier in conventional MTJs. The comparative analysis considered lattice matching, bandgap filtering, and momentum(*k*)-space tunneling compatibility.

**Lattice matching:**

**Fig. S4** exhibits the crystal structures and lattice parameters of Mn_3_SnN and the investigated barriers. BaTiO_3_ and CaTiO_3_ exhibit acceptable mismatches with Mn_3_SnN (2.88% and 0.27%, respectively), comparable to that of SrTiO_3_ (1.67%). MgO, however, has a larger mismatch (~7.89%), exceeding the common theoretical threshold (~5%) for reliable interface modeling.


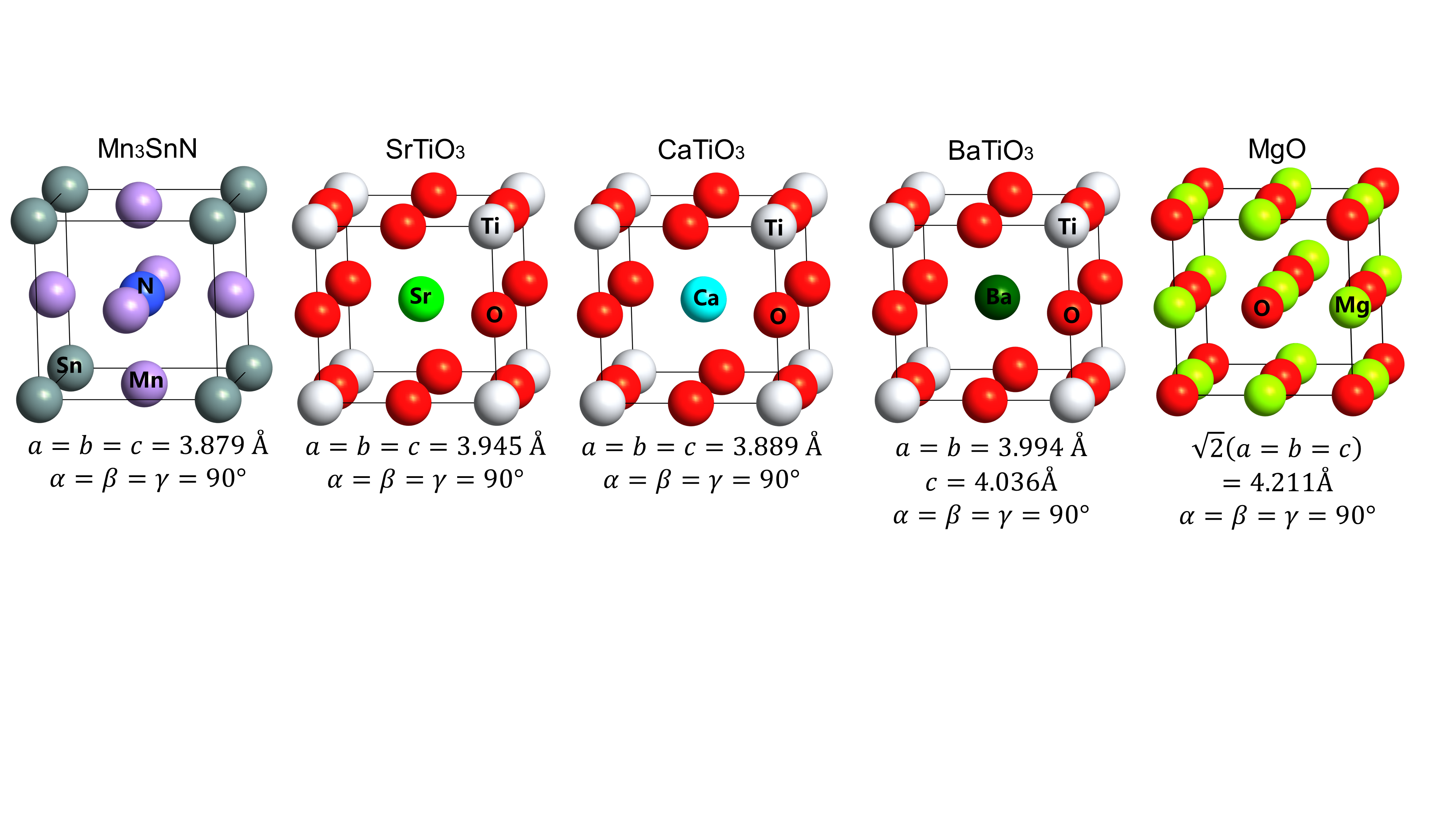


**Figure S4.** Crystal structures and lattice parameters of Mn_3_SnN and four representative tunnel barrier materials—SrTiO_3_, CaTiO_3_, BaTiO_3_, and MgO.

**Bandgap filtering:**

As shown in **Fig. S5**, BaTiO_3_ have a smaller DFT bandgaps (1.8 eV) compared to CaTiO_3_ (~ 2.2 eV) and SrTiO_3_ (~ 2.2 eV), which leads to weaker tunneling selectivity. MgO, with a larger bandgap (~5 eV), offers stronger filtering but suffers from poor interface compatibility.


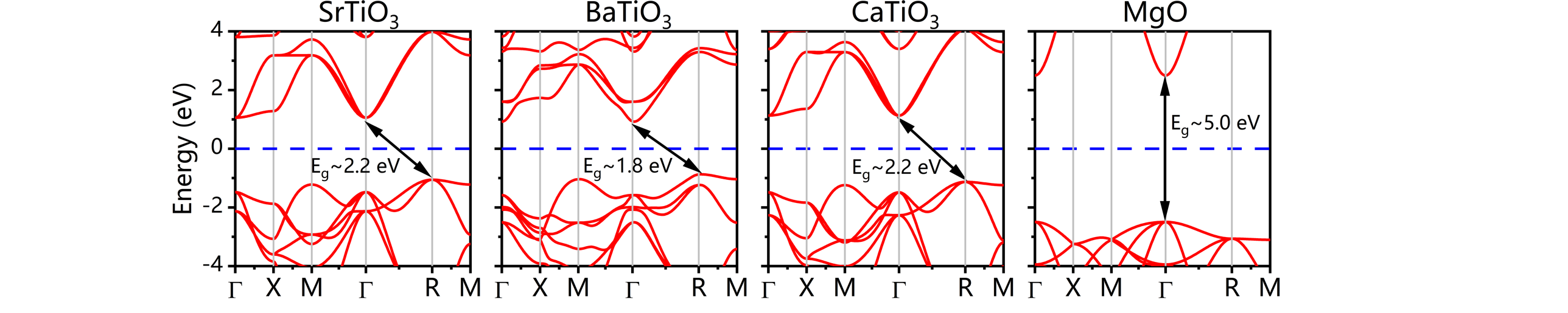


**Figure S5.** Electronic band structures of SrTiO_3_, CaTiO_3_, BaTiO_3_, and MgO. The blue dashed line indicates the Fermi level, and E_g_ denotes the bandgap.

**Momentum(*k*)-space matching and TMR:**

Typically, good matching between the barrier’s *low-decay* regions and the *high-*$p_{k\parallel}$ regions of Mn_3_SnN leads to efficient tunneling of highly spin-polarized states, resulting in higher TMR. **Fig. S6** shows the lowest decay rates ($\kappa_{\boldsymbol{k}_{\boldsymbol{||}}}$) of evanescent states for the investigated barriers (**S6a–S6d**), along with the effective spin polarization $p_{k_{\parallel}}$ = |$\boldsymbol{p}_{\boldsymbol{k}_{\boldsymbol{\parallel}}}$| of Mn_3_SnN in the 2DBZ (**S6e**). Specifically, BaTiO_3_ poorly matches the high-$p_{k\parallel}$ regions of Mn_3_SnN, yielding a low TMR (~37%), while CaTiO_3_ shows better alignment (~200%). SrTiO_3_ shows optimal alignment along the *Γ*–*X* and *Γ*–*M* direction, resulting in a high TMR (~1500%). For MgO, additional calculations using a strained interface model suggest that—if the ~7.89% mismatch could be accommodated experimentally—it could deliver high TMR (~5000%) due to favorable tunneling near the *Γ*-point.


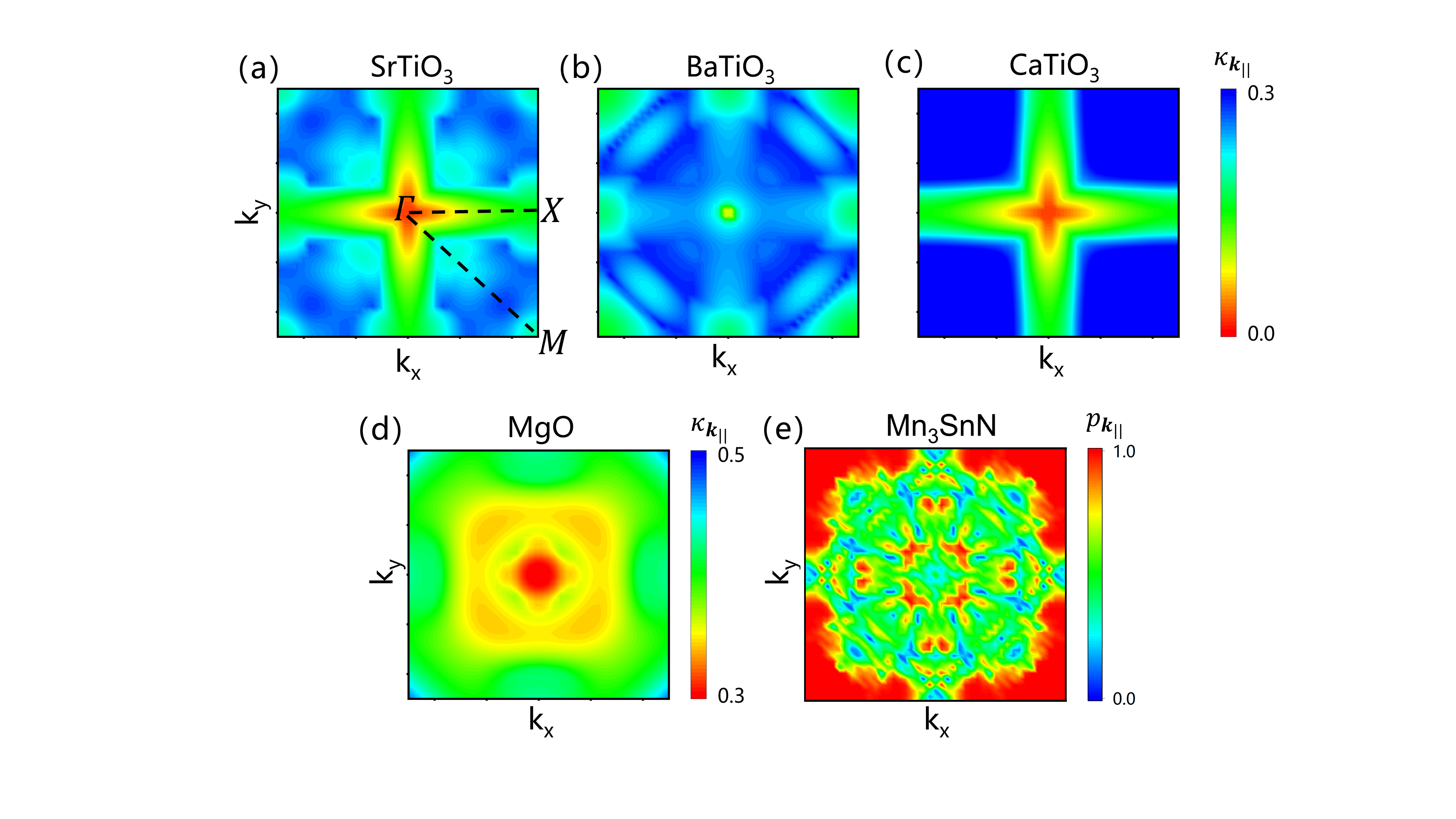


**Figure S6.** The lowest decay rates of evanescent states in the two-dimensional Brillouin zone (2DBZ) for (a) SrTiO_3_, (b) BaTiO_3_, (c) CaTiO_3_, and (d) MgO. Due to different band alignments at the interface with Mn_3_SnN, the decay rates in (a)–(c) are computed near the conduction band minimum of each material and share a color scale ranging from 0.0 to 0.3. In contrast, the decay rate in (d) is calculated at the Fermi level (mid-gap) of MgO, which leads to generally larger values and thus uses a different color scale ranging from 0.3 to 0.5. (e) The magnitude of the effective spin polarization $p_{\boldsymbol{k}_{\parallel}}$ = |$\boldsymbol{p}_{\boldsymbol{k}_{\parallel}}$| in the 2DBZ of Mn_3_SnN.

**Table S2. Summary of the calculated transmission coefficient (*T*), resistance-area (RA), and TMR of Mn_3_SnN AFMTJs with BaTiO_3_, CaTiO_3_, SrTiO_3_ and MgO barrier, respectively.**

| Barrier | Mismatch |  | P | | AP |
| --- | --- | --- | --- | --- | --- |
| **BaTiO_3_** | 2.88% | *T* (*e*^2^/*h*) | 2.05×10^-3^ | | 1.49×10^-3^ |
|  |  | RA (Ω•μm^2^) | 1.90 | | 2.61 |
|  |  | TMR (%) | 38 | | |
| **CaTiO_3_** | 0.27% | *T* (*e*^2^/*h*) | 2.00×10^-2^ | | 5.97×10^-3^ |
|  |  | RA (Ω•μm^2^) | 1.94×10^-1^ | | 6.51×10^-1^ |
|  |  | TMR (%) | 235 | | |
| **SrTiO_3_** | 1.67% | *T* (*e*^2^/*h*) | 5.09×10^-2^ | | 3.14×10^-3^ |
|  |  | RA (Ω•μm^2^) | 7.63×10^-2^ | | 1.24×10^0^ |
|  |  | TMR (%) | 1521 | | |
| **MgO** | 7.89% | *T* (*e*^2^/*h*) | 6.57×10^-5^ | 1.30×10^-6^ | |
|  |  | RA (Ω•μm^2^) | 5.91×10^1^ | 2.97×10^3^ | |
|  |  | TMR (%) | 4954 | | |

Table S2 summarizes of the calculated transmission coefficient (*T*), resistance-area (RA), and TMR of Mn_3_SnN AFMTJs with BaTiO_3_, CaTiO_3_, SrTiO_3_ and MgO barrier. Overall, these findings suggest that SrTiO_3_ provides the most balanced performance in terms of structural compatibility, electronic filtering, and *k*-space tunneling matching. Nonetheless, MgO remains a competitive candidate under appropriate strain engineering.

Furthermore, we note that SrTiO_3_ has already been demonstrated as an effective barrier in AFMTJs, such as in Mn_3_GaN-based systems,^1^ where a theoretical TMR of up to 10,000% was reported—attributed to excellent structural and electronic alignment. However, SrTiO_3_ may not be universally applicable to all non-collinear antiferromagnets; for example, it is less compatible with Mn_3_Sn, which has a hexagonal structure. Therefore, our study emphasizes that both lattice compatibility and momentum-space filtering must be carefully considered when selecting optimal barrier materials for AFMTJs.

**Note 5. Detailed discussion of interfacial defects and lattice mismatch on TMR**

To optimize TMR values, it is therefore critical to simultaneously address surface defects, roughness and crystalline quality of the constituent layers, which demands advanced atomic-scale physical vapor deposition techniques. Another significant challenge arises from lattice mismatch between layers, which induces strain and subsequently alters the material's electronic properties. Notably, recent research has achieved substantial room-temperature TMR in all-antiferromagnetic tunneling junctions based on Mn_3_Pt/MgO/Mn_3_Pt, wherein Mn_3_Pt and Mn_3_SnN possess similar crystal structures. Nevertheless, lattice mismatch issues can be effectively mitigated through either the implementation of suitable buffer layers or the careful selection of materials with matched lattice constants.

**Note 6. Advantages of Mn_3_SnN over Mn_3_Sn for Spintronic Applications**

Mn_3_Sn is a well-studied non-collinear antiferromagnet, and previous studies have provided valuable fundamental insights that have deepened the understanding of non-collinear magnetism and its potential in spintronics. Hexagonal Mn_3_Sn exhibits anomalous Hall conductivity comparable to ferromagnetic materials due to the ferroic ordering of cluster magnetic octupole hosted in its chiral antiferromagnetic state.^2^ The perpendicular modulation of this octupole enables W-generated spin current to completely switch the chiral antiferromagnetic order.^3^

Antiperovskite Mn_3_SnN possesses a cubic crystal structure analogous to Mn_3_Pt, which has demonstrated square anomalous Hall effect (AHE) in Mn_3_Pt(001) film orientation.^4^ Following this, researchers achieved room-temperature AHE in Mn_3_SnN(001) films.^5^ Through process optimization, Mn_3_SnN has potential to exhibit near-square AHE similar to recently discovered CrSb.^6^ This room-temperature AHE property suggests Mn_3_SnN(001) could be effectively controlled using moderate magnetic fields.

Compared to Mn_3_Sn, Mn_3_SnN is not simply a N-doped variant; rather, the incorporation of N leads to a complete transformation of the crystal structure, resulting in significant differences in material properties. While both Mn_3_Sn and Mn_3_SnN are non-collinear AFMs exhibiting intriguing spin textures, Mn_3_SnN offers several advantages that make it more suitable for integration into spintronic devices, particularly AFMTJs.

First, Mn_3_SnN, with its cubic anti-perovskite structure, is structurally more compatible with common oxide barriers such as SrTiO_3_ (cubic perovskite) and MgO (rock-salt cubic), compared to the hexagonal structure of Mn_3_Sn. Although the lattice mismatch between Mn_3_SnN and MgO is relatively large, it is still more favorable than that between MgO and hexagonal Mn_3_Sn, highlighting the advantage of Mn_3_SnN for device integration.

Second, Mn_3_SnN exhibits a relatively high Néel temperature (~475 K),^5,7–9^ providing enhanced thermal stability and robust antiferromagnetic order under ambient conditions. In comparison, Mn_3_Sn, with a lower Néel temperature (~420 K),^10^ offers reduced thermal robustness, further underscoring the advantage of Mn_3_SnN for practical applications.

Third, current theoretical calculations show that Mn_3_SnN-based AFMTJ exhibits significantly higher TMR compared to the Mn_3_Sn counterpart. In our work, the TMR of the Mn_3_SnN-based AFMTJ reaches up to 1500%, nearly five times greater than the maximum theoretical value of 300% reported for Mn_3_Sn^10^. This comparison indicates that Mn_3_SnN-based AFMTJs offer superior distinction between parallel and antiparallel resistance states ("0–1" readout), which is of great importance for practical spintronic device applications.

Therefore, Mn_3_SnN offers several distinct advantages for practical device applications, including better structural compatibility with conventional barrier materials, higher thermal stability, and a significantly larger theoretically predicted TMR. These features make Mn_3_SnN a more promising candidate than Mn_3_Sn for next-generation, energy-efficient, and scalable AFM-based spintronic devices.

**Note 7. Schematic diagram of experimentally implemented multi-state TMR**


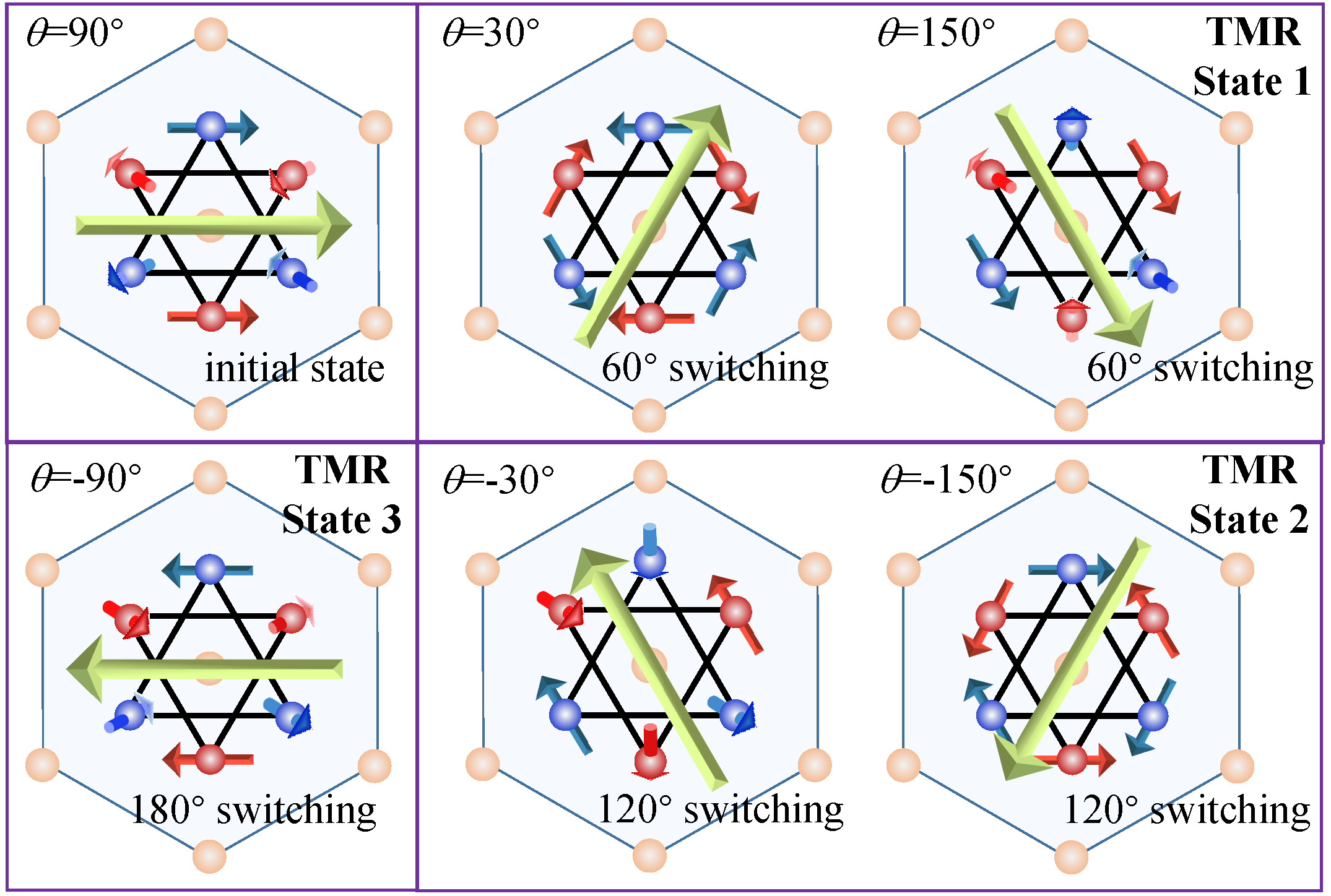


**Figure S7.** Spin structure of Mn_3_SnN kagome (111) plane. The blue and red circles (orange circles) represent Mn (Sn) atoms. The magnetic moments of the triangular Mn atoms are indicated by red and blue arrows, respectively. Note that the N atoms are obscured by the Sn atoms after projection. The green arrow indicates the magnetic octupole moment direction. *θ* is defined as the azimuthal angle of the magnetic octopole moment in the (111) kagome plane. Magnetic octupole moment has six different stable directions: *θ*=±30°, ±90° and ±150°.

**Reference**

1. Gurung, G., Elekhtiar, M., Luo, Q.-Q., Shao, D.-F. & Tsymbal, E. Y. Nearly perfect spin polarization of noncollinear antiferromagnets. *Nat. Commun.* **15**, 10242 (2024).

2. Nakatsuji, S., Kiyohara, N. & Higo, T. Large anomalous Hall effect in a non-collinear antiferromagnet at room temperature. *Nature* **527**, 212–215 (2015).

3. Higo, T. *et al.* Perpendicular full switching of chiral antiferromagnetic order by current. *Nature* **607**, 474–479 (2022).

4. Liu, Z. Q. *et al.* Electrical switching of the topological anomalous Hall effect in a non-collinear antiferromagnet above room temperature. *Nat. Electron.* **1**, 172–177 (2018).

5. You, Y. *et al.* Room temperature anomalous Hall effect in antiferromagnetic Mn_3_SnN films. *Appl. Phys. Lett.* **117**, 222404 (2020).

6. Zhou, Z. *et al.* Manipulation of the altermagnetic order in CrSb via crystal symmetry. *Nature* **638**, 645–650 (2025).

7. Takenaka, K. *et al.* Magnetovolume effects in manganese nitrides with antiperovskite structure. *Sci. Technol. Adv. Mater.* **15**, 015009 (2014).

8. Feng, W. J. *et al.* Structural, magnetic and transport properties of Mn3.1Sn0.9 and Mn_3.1_Sn_0.9_N compounds. *J. Alloys Compd.* **437**, 27–33 (2007).

9. Zemen, J., Gercsi, Z. & Sandeman, K. G. Piezomagnetism as a counterpart of the magnetovolume effect in magnetically frustrated Mn-based antiperovskite nitrides. *Phys. Rev. B* **96**, 024451 (2017).

10. Dong, J. *et al.* Tunneling Magnetoresistance in Noncollinear Antiferromagnetic Tunnel Junctions. *Phys. Rev. Lett.* **128**, 197201 (2022).
